# The muscle satellite cell at 50: the formative years

**DOI:** 10.1186/2044-5040-1-28

**Published:** 2011-08-17

**Authors:** Juergen Scharner, Peter S Zammit

**Affiliations:** 1 Randall Division of Cell and Molecular Biophysics, King's College London, London, SE1 1UL, UK

## Abstract

In February 1961, Alexander Mauro described a cell 'wedged' between the plasma membrane of the muscle fibre and the surrounding basement membrane. He postulated that it could be a dormant myoblast, poised to repair muscle when needed. In the same month, Bernard Katz also reported a cell in a similar location on muscle spindles, suggesting that it was associated with development and growth of intrafusal muscle fibres. Both Mauro and Katz used the term 'satellite cell' in relation to their discoveries. Today, the muscle satellite cell is widely accepted as the resident stem cell of skeletal muscle, supplying myoblasts for growth, homeostasis and repair.

Since 2011 marks both the 50th anniversary of the discovery of the satellite cell, and the launch of *Skeletal Muscle*, it seems an opportune moment to summarise the seminal events in the history of research into muscle regeneration. We start with the 19th-century pioneers who showed that muscle had a regenerative capacity, through to the descriptions from the mid-20th century of the underlying cellular mechanisms. The journey of the satellite cell from electron microscope curio, to its gradual acceptance as a *bona fide *myoblast precursor, is then charted: work that provided the foundations for our understanding of the role of the satellite cell. Finally, the rapid progress in the age of molecular biology is briefly discussed, and some ongoing debates on satellite cell function highlighted.

## Introduction

Skeletal muscle accounts for a sizable proportion of body weight, being just over 30% for a typical women, and around 38% for men (e.g. [1]). The basic unit of skeletal muscle is the myofibre: a syncytial cell packed with myofibrils, containing the sarcomeres that generate force by contraction. In vertebrates, each myofibre is controlled by many (usually hundreds) of myonuclei, which in mammals are considered post mitotic. During postnatal growth, new myonuclei are supplied by muscle satellite cells: resident stem cells located on the surface of a myofibre. Satellite cells then become mitotically quiescent in mature muscle, but remain able to be recruited to provide myoblasts for muscle hypertrophy and repair.

**Figure 1:**
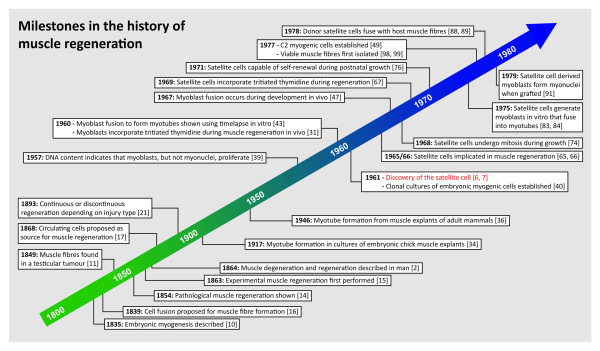
**Timeline of some seminal events in the history of muscle regeneration**.

That muscle is capable of regeneration was first shown in the 1860s [2-5], but almost a century elapsed before the satellite cell was discovered. Here, we first document the seminal findings of the 19th and early 20th century pioneers that described muscle regeneration, through to the work of the 1950s and 1960s that revealed the underlying cellular mechanisms. During this latter period, the satellite cell materialised and we chronicle its journey from electron microscope curio to its general acknowledgement as a muscle precursor cell, providing myoblasts for postnatal muscle growth and regeneration (Figure 1). We end by briefly highlighting how this fundamental work provided the basis for rapid progress in the age of molecular biology, and discuss some ongoing debates in satellite cell function.

### Discovery of the muscle satellite cell

**Figure 2:**
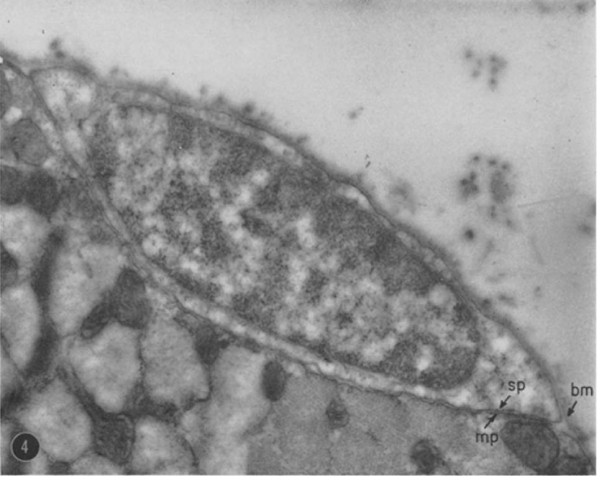
**The first mammalian satellite cell**. The electron micrograph of a mammalian satellite cell from Mauro's 1961 paper [6]. Described in his own words: 'Transverse section of a skeletal muscle fiber from the rat sartorius, furnished by courtesy of Dr. G. Palade. As in Fig. 9, the apposing plasma membranes of the satellite cell *(sp) *and the muscle cell *(mp) *are seen at the inner border of the satellite cell. The basement membrane *(bm) *can be seen extending over the "gap" between the plasma membrane of the muscle cell and the satellite cell. Methacrylate embedding. Stained with PbOH. × 22,000'. ^© ^The Rockefeller University Press. *J Biophys Biochem Cytol *1961, **9:**493-495.

In February 1961, two papers were published that identified a cell residing on the surface of skeletal muscle fibres [6,7]. In the more famous of the pair, Alexander Mauro used electron microscopy to describe a cell with a striking paucity of cytoplasm relative to its nucleus 'wedged between the plasma membrane of the muscle fibre and basement membrane' in the tibialis anticus muscle of the frog [6]. 'Alerting' other investigators resulted in similar cells also being found on myofibres in the sartorius and ileofibularis of the frog and in rat tongue and sartorius muscle (Figure 2). Mauro states that these cells intimately associated with muscle fibres 'we have chosen to call satellite cells' (anatomical name: myosatellitocytus; [8]), uniting the defining anatomical location with the name. Interestingly, René Couteaux had used the term 'éléments satellites' to describe 'undifferentiated elements' on the surface of myotubes during his 1941 description of secondary myogenesis [9]. Even at its discovery, a role as a muscle precursor cell was postulated, with Mauro presciently speculating that 'satellite cells are merely dormant myoblasts that failed to fuse with other myoblasts and are ready to recapitulate the embryonic development of skeletal muscle fiber when the main multinucleate cell is damaged' [6]. Importantly, cardiac muscle did not contain satellite cells, with Mauro commenting that 'It is exciting to speculate whether the apparent inability of cardiac muscle cells to regenerate is related to the absence of satellite cells'. Coincidentally, in February 1961 in a paper exploring the innervation of the frog muscle spindle, Bernard Katz also reported cells on the surface of intrafusal muscle fibres, mentioning that 'the surface of many muscle fibres is invested here and there with hypectolemmal satellite cells'. Katz also speculated that satellite cells were associated with development and growth of muscle spindles [7].

### A brief history of skeletal muscle regeneration

So what was known about muscle regeneration when the satellite cell was discovered? Archiving of journals and books is moving apace, with many of the significant papers on muscle regeneration now available online. This enables us to first outline the seminal studies of the 19th and early 20th century that described the basics of muscle regeneration (but note that in the early years, muscle regenerated largely in German!).

In 1835, Gabriel Gustav Valentin examined muscle formation during embryogenesis and described how corpuscles of the primordial mass align and fuse to form a transparent mass, the muscle fibre [10]. This view was adopted and refined by Theodor Schwann (cofounder of the cell theory) in 1839, who noted that 'every primitive muscle bundle is a secondary cell formed by fusion of primary nuclei containing round cells that were aligned in a row', although the formal proof that muscle fibres arose from cell fusion took another 120 years or so.

Early reports of non-developmental myogenesis were in disease, where Baron Carl von Rokitansky described 'accidental formation of new cross striated muscle fibres' in a testicular tumour in 1849 [11], with similar observations in tumours by Rudolf Virchow [12] and Theodor Billroth [13]. Pathological formation of myofibres within skeletal muscle was described by Carl Otto Weber in a hypertrophic tongue. He inventively compared these newly-formed muscle fibres with developing muscle from 4-5-month-old human embryos and noted a similarity in size and morphology [14]. In 1863, Weber followed his studies in man by examining muscle injuries in rabbits, observing new muscle fibre formation in scar tissue, and probably introducing animal models to the study of regenerative myogenesis [4,5,15].

1864 saw Friedrich Albert von Zenker publish his analysis of the pathological changes in voluntary musculature in post mortem samples from victims of abdominal typhoid fever [2]. Although focusing mainly on characterising muscle degeneration, Zenker also noted extensive proliferation of cells in place of degenerated fibres and formation of new muscle tissue in the healing musculature. Inspired by Zenker's work, Wilhelm Waldeyer produced the first extensive experimental study on muscle regeneration in 1865 [3]. In addition to studying victims of abdominal typhoid fever, Waldeyer also investigated muscle injuries in frogs, guinea pigs and rabbits. He observed 'Muskelkörperchen' (muscle corpuscles) stuffed into spindle shaped tubes, which he argued were muscle cells growing within the sarcolemma of degenerating muscle fibres. Unfortunately he could not link these cells to muscle regeneration and therefore concluded, in line with Zenker, that cells from the connective tissue were the origin of these new muscle fibres. Weber, however, argued that new muscle fibres actually originate from proliferating 'muscle corpuscles' rather than cells from connective tissue [4,5], following the same process as described by Valentin [10] and Schwann [16] during developmental myogenesis.

The source of new muscle remained a subject of debate. Maslowsky in 1868 [17] and Erbkam in 1880 [18] thought that new muscle fibres derived from circulating leucocytes, so called 'Wanderzellen'. Budge and Weismann (cited in [3] and [19]) claimed instead, that the source of new muscle fibres was from the splitting of old myofibres, while Neumann 1868 [20] and others (cited in [19]) proposed that the 'contractile substance' of old fibres 'sprout' and form young fibres that then integrate into the muscle tissue. From these various observations/hypotheses emerged two theories that would occupy researchers for the next century or so: in 'discontinuous' or 'embryonic' regeneration, new muscle fibres were formed from cells fusing together, in a process akin to developmental myogenesis, while 'continuous' regeneration involved myofibres arising as multinucleated extensions or outgrowths (buds) from surviving muscle fibres.

Along with Waldeyer and Weber, the other towering figure of 19th century muscle regeneration research is Rudolf Volkmann [21]. In the first truly comprehensive study of muscle regeneration, Volkmann used multiple species (including man, dog, pig, rabbit and guinea pig) combined with various types of injury, performing 104 experiments and analysing more than 1,700 stained tissue sections. Ingeniously, he grouped his specimens into two categories according to the type of injury: either those that primarily affect the contractile substance but preserve the sarcolemma and connective tissue (cryodamage, burns and typhoid fever), or those that induce necrosis and the loss of 'sarcolemma tubes' and cause severe gaps in the connective tissue (constrictions, incisions, excisions, cauterisations, severe cryodamage and burns). He concluded that muscle regeneration always originates from the nuclei of old muscle fibres and depending on the injury, is either continuous, discontinuous or both [21].

The early years of the 20th century saw *in vivo *research becoming more sophisticated. While those such as Julius Elson favoured discontinuous regeneration [22], a consensus was building around continuous regeneration, for example, W. Gilbert Millar [23]. Of particular note was a series of papers by Wilfred Le Gros Clark [24-26] detailing just how effective regeneration was in rabbit and rat after muscle grafting, crush injury or ischaemia. Le Gros Clark concluded that continuous regeneration was the mechanism. Furthermore, he felt that the increase in myonuclear number during muscle regeneration involved 'amitosis', whereby existing myonuclei synthesised DNA and then split to produce two new nuclei, but without any accompanying cytoplasmic division [24]. The remarkable regenerative ability of skeletal muscle was further shown in a series of studies by Studitsky in the 1950s and 1960s, where even mincing rodent or bird muscle to a fine slurry before grafting back into its original location still resulted in regeneration to form a new functional muscle (e.g. [27], reviewed in [28]).

### Myoblasts, but not myonuclei, undergo cell division during muscle regeneration

Labelling newly synthesised DNA with tritiated thymidine was developed by Herbert Taylor [29,30] and was a revolutionary technique to study mitosis, that could also be used to label cells for lineage tracing. Tritiated thymidine was first used to study muscle regeneration in a seminal paper by Sharon Bintliff and Bruce Walker in 1960 [31], and the technique would figure largely in muscle research until the mid 1980s.

**Figure 3:**
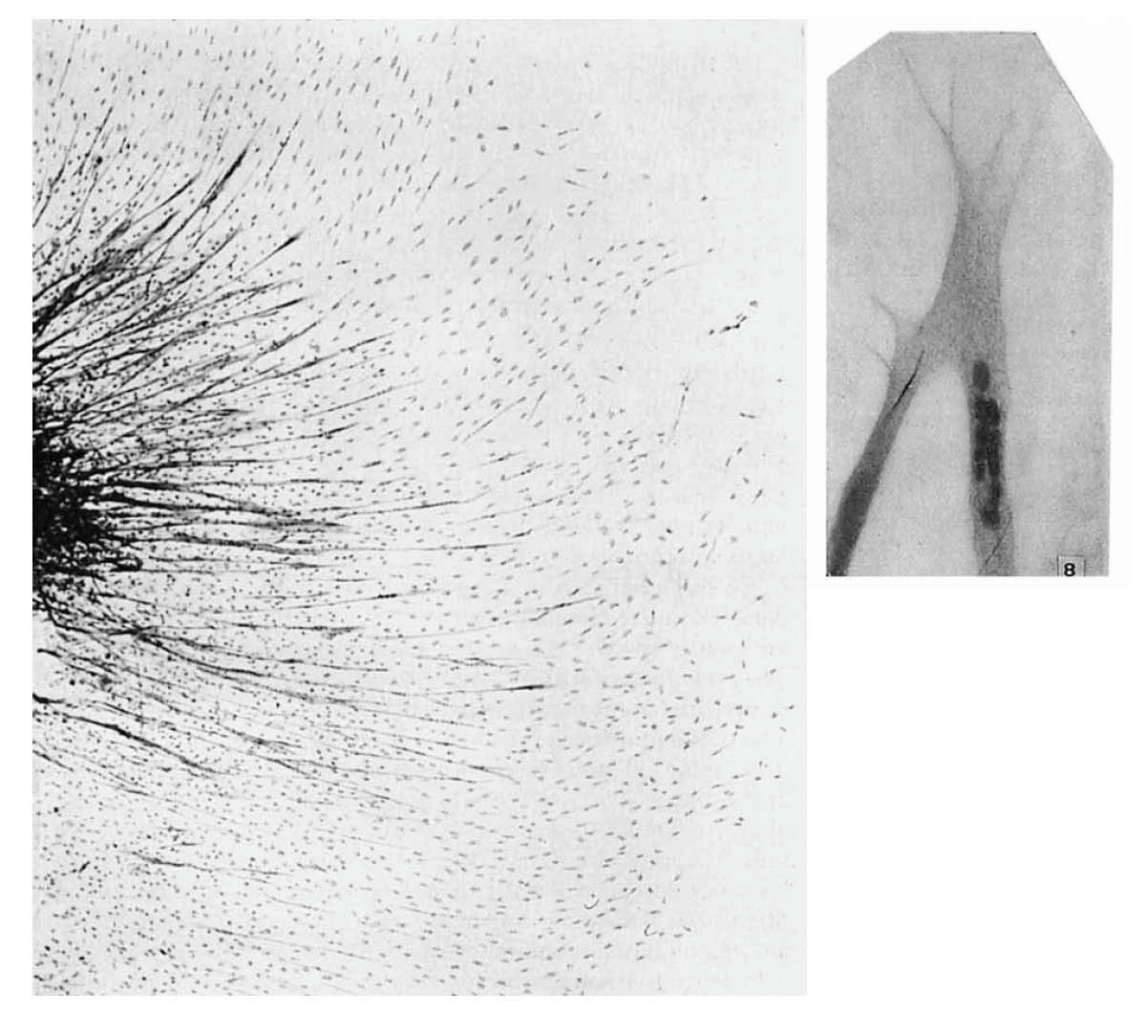
**Images of cultivated chick embryonic muscle from 1917**. Two images of chick muscle in culture from Lewis and Lewis, 1917 [34]. The image on the left is outgrowth from leg muscle of a 7-day-old chick embryo cultivated in half Locke's solution, half bouillon plus 0.5% dextrose for 48 hours. The preparation was fixed in osmic acid vapour and a Benda stain used. Lewis and Lewis describe it thus: 'Somewhat different character of muscle outgrowth from an explanted piece of the same leg and cultivated in the same way as in figure 1. The enlarged protoplasmic ends are not so abundant. There are many isolated muscle fibers and myoblasts among the mesenchyme cells. × 100' (originally figure 2 of Lewis and Lewis, 1917 [34]). The image on the right is of a 'myotube' from an explanted piece of leg of an 8-day-old chick embryo, fixed in osmic acid vapour, and stained with iron haematoxylin at × 525 (originally figure 8 of Lewis and Lewis, 1917 [34]). ^© ^John Wiley & Sons, Inc. *Am J Anat *1917, **2:**169-194. This material is reproduced with permission of John Wiley & Sons, Inc.

**Figure 4:**
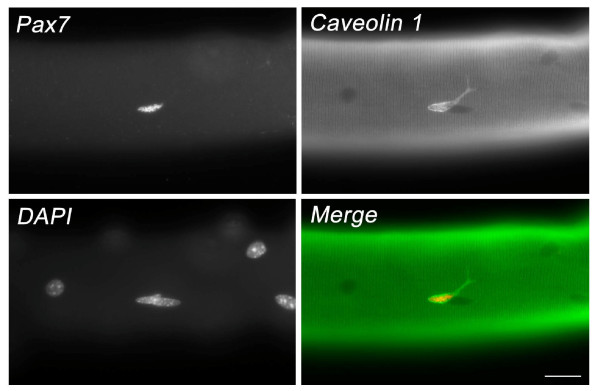
**A mouse satellite cell in its niche on an isolated myofibre**. A myofibre isolated from the extensor digitorum longus muscle of an adult mouse. The associated quiescent satellite cell was coimmunostained for Pax7 and caveolin 1. Nuclei were counterstained with 4',6-diamidino-2-phenylindole (DAPI), revealing the location of the myonuclei in the myofibre. Scale bar = 20 μm.

Shortly after pulsing regenerating murine muscle with tritiated thymidine, Bintliff and Walker noted that only mononucleated cells contained the label, and so were actively synthesising DNA. The radioactive signal only became detectable in nuclei of newly regenerated myotubes 4 days after the pulse. Importantly though, myotube nuclei did not label directly if the pulse was administered later in the regenerative process. Thus mononucleated cells were able to synthesise DNA, and the myoblasts amongst them then retained the label after they differentiated into myotubes. Holding no punches, Bintliff and Walker concluded 'However, the evidence derived through microspectrophotometry (Lash *et al*., 57) and radioautography (present report) indicating limited mitotic proliferation prior to myotube formation and no nuclear proliferation thereafter, demonstrates the irrelevance of theories about amitosis for the understanding of muscle regeneration and should help to emphasise the fallacy of adhering to the theory of amitosis as long as it is based on such trivial evidence as the wrinkling of nuclei'. Interestingly, they also noted silver grains over some muscle nuclei in their normal positions on the periphery of muscle fibres in the area of the wound, leading them to conclude that the new nuclei were derived by dedifferentiation of myonuclei [31]: however these are probably the first images of activated satellite cells *in vivo*. The following year, Paul Pietsch used colchicine to disrupt mitosis and cause the accumulation of mitotic figures during muscle regeneration in mice and reported that 'Though a given microscopic field might possess many arrested mitotic figures (Figures 3 and 4), cell division was never found within a muscle tube. Occasionally, a dividing cell was in close proximity with a muscle fiber and appeared to be within it. Examination at a magnification of 970 × revealed, always, that these cells were on, rather than within the muscle fibers' [32].

### Multinucleated muscle fibres are formed by the fusion of myoblasts

Another revolutionary technique was tissue and cell culture, which was developed in the early years of the 20th century (reviewed in [33]). The first comprehensive description of muscle formation *in vitro *was published in 1917 [34]. Warren Lewis and Margaret Lewis cultured portions of embryonic chick muscle and were able to observe outgrowths from the end of cut muscle fibres, which they attributed to dedifferentiation of muscle fibres. However they also noted many mesenchymal cells had mitotic figures, and that 'There are many isolated muscle fibers and myoblasts among the mesenchyme cells' (Figure 3; images from Lewis and Lewis [34]). These isolated muscle fibers would now be termed myotubes, but the word 'myotube' (miotubas) and 'myofibre' (miofibras) had only just been coined by Jorge Francisco Tello in the same year [35]. In 1946, Irene Pogogeff and Margaret Murray cultured human and rat muscle fragments isolated from adult [36], and so first documented cultured myotubes derived from previously quiescent satellite cells.

Immunostaining was applied to muscle in 1956 by Howard Holtzer's group [37]. The following year, while studying myofibril assembly during early myogenesis, Holtzer, John Marshall and Henry Finck noted that 'Multinucleated myoblasts appear first during the 4th day. It is our impression that each forms by the fusion of a spindle-shaped mononucleated myoblast with several mononucleated mesenchyme-like cells, the presumptive myoblasts, but this subject will require further analysis' [38], which promptly followed. Using his own technique of microspectrophotometric measurement of DNA content using Fuelgen staining of single nuclei, Hewson Swift (together with James Lash and Holtzer), concluded that nuclei of regenerated mouse muscle were 99% diploid [39]. However approximately 10% of mononucleated cells had more DNA than the diploid state, indicating DNA synthesis in preparation for cell division, with such cells occasionally containing mitotic figures. Since the nuclear morphology of these mononucleated cells was similar to that of nuclei in myotubes, the authors tentatively proposed 'that the accumulation of the centrally placed nuclei is the result of mobilisation, not extensive proliferation within the regenerating myotube' [39].

Trypsinising tissue to release individual cells transformed cell culture, allowing monolayer cell culture to be developed (reviewed in [33]). Employing such techniques, Irwin Konisberg found that chick myoblasts could proliferate and give rise to clones of differing size. Cells in some clones then differentiated to produce multinucleated myotubes [40,41]. Konigsberg proposed that such myotubes arose via cell fusion [42], and timelapse photography by Charles Capers [43] and then William Cooper and Konigsberg [44] documented individual myoblasts fusing both together, and to established myotubes. Combining monolayer culture with tritiated thymidine pulsing, Frank Stockdale and Holtzer showed that chick myoblasts were able to incorporate label when proliferating, whereas the nuclei of myotubes never did. However, when labelled myoblasts underwent differentiation, tritiated thymidine then appeared in myotube nuclei [45]. These results were consistent with both the observations that mitotic figures were only ever detected in myoblasts [44,45], and that myotube nuclei were relatively unaffected by inhibition of DNA synthesis using nitrogen mustard, whereas mononucleated cells died [46]. Beatrice Mintz and Wilber Baker later confirmed that multinucleated myotubes also formed by cell fusion *in vivo*. Using mouse embryos mosaic for two different isozyme subunits, they showed that skeletal muscle contained hybrid enzyme, meaning that cells expressing the two distinct isoenzyme subunits had fused together during development and so shared a common cytoplasm [47]. Collectively, these observations established that myotubes form and grow by fusion of mononucleated cells, as originally proposed by Valentin [10] and Schwann [16] in the 1830s.

### Establishment of myogenic cell lines

Cloning myogenic cells also led to the establishment of permanent muscle cell lines, which provide a ready source of myogenic cells and remain the workhorse of many studies to this day. David Yaffe first generated the L6, and then the permanent L8, myogenic lines from newborn rat in 1968 [48]. Later, while trying to generate lines from dystrophic adult *dy *mice, Yaffe and Ora Saxel (now Ora Fuchs) produced a control line (C_2_) from injured thigh muscle of 2-month-old C3H mice [49]: likely derived from the progeny of activated satellite cells. Helen Blau and colleagues recloned C_2 _cells and expanded them into the C2C12 cell line [50]. Exploring the phenotypes of primary muscle colony forming cells from early and late stage embryos and from adults, Stephen Hauschka's group found that the phenotypes differed and were heritable. Hauschka established many permanent clonally derived lines, including MM14 from adult mouse muscle [51], showing that single isolated cells from adult skeletal muscle gave rise to muscle colonies that could be greatly expanded using fibroblast growth factor, prior to their spontaneous transformation into myogenic cell lines [52].

### The muscle satellite cell enters the picture

After the initial description of satellite cells in frog and rat by Mauro and Katz [6,7], cells with a similar morphology were also noted in a sublaminal location on muscle fibres in man, cat, dog [53], mouse and fruit bat [54,55]. The lack of continuity between the satellite cell cytoplasm and that of the muscle fibre was confirmed, since the satellite cell was shown to be resistant to osmotic swelling of the associated myofibre [54]. A generalised morphological description emerged of a bipolar cell with a nucleus containing abundant heterochromatin, surrounded by a thin rim of perinuclear cytoplasm with few mitochondria, little rough endoplasmic reticulum, undeveloped Golgi apparatus and no myofilaments (Figure 2; reviewed in [8]). Satellite cells were quickly shown to be rare in healthy uninjured adult muscle, accounting for 4.8% to 5.8% of muscle fibre nuclei in rat and mouse [55] to approximately 10% in adult bat web muscles [54]. However, satellite cell frequency was later found to vary between different muscles, being higher in the rat 'slow' soleus muscle, than in the 'fast' extensor digitorum longus [56]. Furthermore, although generally distributed along the length of the myofibre, satellite cells were seen to concentrate at the neuromuscular junction in some muscles, such as the soleus [57].

### Satellite cells are implicated in muscle regeneration

Satellite cells were not universally accepted as a source of myoblasts for muscle regeneration, with the topic remaining controversial for many years. This was in part due to the requirement of electron microscopy for the confirmation of satellite cell identity, which was laborious and limited the range and scale of experiments. While early electron microscopic studies of muscle regeneration clearly described myoblasts, they did not make a connection to the recently described satellite cell [58,59]. Indeed, the view that myoblasts originated from myonuclear dedifferentiation after trauma, in a manner similar to that proposed for salamander [60,61], remained widely accepted. For example, while Walker found that new myonuclei largely arose from nuclei within the muscle fibres, and not from the connective tissue, he attributed their provenance to dedifferentiation of myonuclei [62]. It was proposed that surviving myonuclei with a surrounding of cytoplasm became encased in a plasma membrane, thus providing the source of satellite cells and their myoblast progeny, which then multiplied by mitotic division during both regeneration [63] and denervation [64].

The first examination of satellite cells in mammalian muscle regeneration was in 1965 by Saiyid Shafiq and Michael Gorycki [65]. They described undifferentiated cells with a distinct similarity in morphology to 'young' myoblasts, which were often located on the periphery of damaged sections of mouse muscle fibres, especially obvious where the myofibre had retracted to leave a clear stretch of basal lamina [65]. Thus satellite cells endured myofibre trauma and were more abundant in areas of muscle damage than in uninjured muscle. In the following year, David Allbrook, with John Church and R. Noronha, studied satellite cells after crush injury to the small web muscles of the East African fruit bat, *Eidolon helvum *[66]. They reported that satellite cells survived in their usual sublaminal position, despite destruction of myonuclei and syncytium around them. Satellite cells then became rare and eventually absent in regions of maximal damage. However, their disappearance coincided with the emergence of myoblasts that later contained mitotic figures and subsequently fused into myotubes. Importantly, satellite cells reappeared on myotubes [66], indicating that the satellite cell pool had been replenished. As the authors elegantly state 'The combined evidence supports the concept that the satellite cells of skeletal muscle are true reserve cells, capable of transformation, following injury, into myoblasts which by mitotic division give rise to new muscle-fibres. Waldeyer's ' Muskelkörperchen' of 100 years ago were surely the muscle satellite cells of electron microscopy' [66]. That *bona fide *mouse satellite cells could synthesize DNA in regenerating muscle was shown by Michel Reznik towards the end of the decade using electron microscopy [67], resolving the identity of at least some of the labelled cells recorded by Bintliff and Walker 9 years earlier [31].

Satellite cells were also investigated in muscle disorders, where it had been shown that a small proportion of myotubes in dystrophic *dy *mice incorporated tritiated thymidine, indicating active muscle regeneration [68]. Muscle biopsies from patients with progressive muscular dystrophy, Duchenne muscular dystrophy or polymyositis contained more satellite cells associated with damaged and regenerating myofibres. These satellite cells had a morphology that indicated that they were not quiescent, since they contained comparatively large amounts of cytoplasm with numerous ribosomes attached to rough endoplasmic reticulum [69,70]. Therefore, after both physical and pathological injury, cells in the satellite cell location exhibited a morphology similar to that of myoblasts, and so appeared to be their precursors.

### The role of satellite cells during postnatal muscle growth

In parallel to studies on regeneration in adult, the satellite cell was also being investigated as a source of myoblasts for muscle growth. In a limited study of 1898, Benedetto Morpurgo had found that the adult number of muscle fibres in rat was reached perinatally, and muscle later grew by addition of contractile substance to each fibre, while the number of nuclei remained unchanged [71]. Undifferentiated, spindle shaped mitotic elements located between the mature myofibres were also described, which Morpurgo argued were the source of new myofibres [71]. In 1964, M. Enesco and Della Puddy conducted a more systematic examination in rat and found that muscle grew by both an increase in myofibre size, and the addition of more nuclei, during postnatal growth, but that the number of myofibres did not change [72]. In an accompanying paper with Charles Leblond, it was shown that rare nuclei beneath the basal lamina of the growing muscle fibre incorporated tritiated thymidine or contained mitotic figures after colchicine treatment. The authors hypothesised that the cells synthesising DNA and undergoing mitosis were satellite cells, but did not use electron microscopy to verify their identity [73]. Harunori Ishikawa noted that satellite cells in developing human and mammalian muscle had a more 'active' appearance (Golgi and well developed granular endoplasmic reticulum) than in mature muscle [53]. That it was the satellite cells that incorporated tritiated thymidine during muscle growth was validated by Mauro, Shafiq and Gorycki using electron microscopy in 1968 [74].

Examining the dynamics of nuclear accretion in myofibres during growth, Francis Moss and Leblond found that satellite cells incorporated tritiated thymidine in growing rats when examined shortly after a pulse, with label not appearing in myonuclei until at least 24 hours later [75,76]. Although other cell types were presumably also labelled, it strongly indicated that satellite cells were a source of myonuclei for muscle growth. Importantly though, this study also estimated that not all satellite cell progeny became myonuclei after each division, indicating that some divisions must have generated both a myonucleus and a satellite cell [76]. Thus the concept of satellite cells being able to self-renew was introduced.

Satellite cell numbers were revealed to drop during maturation, with approximately 30% to 35% of muscle nuclei being satellite cells in perinatal rat, which fell to approximately 10% by postnatal day 28, and to < 5% in adult [77], with a similar trend reported in mouse [54,78]. Incorporation of tritiated thymidine in muscles from older rats was extremely low [72,76], with Edward Schultz showing that even after 9 days continuous administration, satellite cells in 4-month-old mice did not label [79]. As muscle reached maturity, satellite cell morphology also changed, with fewer ribosomes present and rough endoplasmic reticulum greatly reduced: indicative of reduced metabolic activity [80]. Thus the collective evidence favoured satellite cell division to supply myoblasts during postnatal growth, before satellite cells became mitotically quiescent in mature muscle.

### Satellite cells prove their myogenic credentials

The debate about the source of myoblasts for muscle regeneration continued, with Bruce Carlson commenting in 1973 that 'Although some attempts have been made to equate 'activated satellite cells' with presumptive myoblasts, it seems best to wait until the relationship between satellite cells and regeneration is unequivocally proven or disproven before including or excluding satellite cells or their activated stages in the same series as recognisable myoblastic cells' [81]. Two studies followed in 1975, using cultures of myofibres isolated with their associated satellite cells [83,84], which went a long way to prove this relationship.

Pogogeff and Murray had shown that culture of adult muscle could lead to the production of new myotubes [36]. These myogenic precursors normally resided on the myofibre surface, as Richard Bischoff found that only enzymes that fragmented the basal lamina could liberate myogenic cells [82]. Bischoff then physically peeled myofibres from adult rat muscle and observed small mononucleated cells within clear stretches of basal lamina that remained after segmental degeneration. After a lag period, these cells began to proliferate to form clones, before multinucleated myotubes appeared within these surviving areas of basal lamina [83]. Konigsberg's group also physically separated fibre fragments from juvenile quail muscle and saw isolated bulges containing satellite cells appearing during culture. Perhaps due to the immaturity of the basal lamina, these cells escaped the myofibre and proliferated on the cell culture substrate to form colonies that could differentiate into myotubes [84].

### Satellite cell-mediated myogenesis *in vivo*

Muscle transplantation as a tool to study regeneration was employed by Volkmann [21], and refined by, amongst others, Elson [22] and Studitsky [27]. Studies on muscle regeneration now combined transplantation with lineage tracing, to determine the fate of grafted tissue/cells. Joseph Neerunjun and Victor Dubowitz first used tritiated thymidine to follow cell fate in grafted mouse muscle in 1975 [85]. By only giving a short pulse of tritiated thymidine to growing muscle, Stichová and colleagues labelled just donor satellite cells, and after free grafting of the muscle, detected labelled nuclei on the periphery of regenerated myofibres in the host [86]. Mikel Snow further refined these protocols in a couple of seminal papers [87,88]. Regular pulsing rats during both the embryonic and neonatal periods resulted in myonuclei, but not satellite cells, containing tritiated thymidine when analysed 1 month later. If such labelled muscle was then minced and grafted, tritiated thymidine was not found in 'viable nuclei' (assumed satellite cells) in the host after 8 and 16 hours. Pulsing the donor an hour before death, however, resulted in only satellite cells incorporating tritiated thymidine. When this muscle was minced and grafted, label was only present in 'viable nuclei' 8 and 16 hours later [87], while after 5-7 days of *in vivo *regeneration, rare labelled nuclei were present in newly formed myotubes [88]. Similarly, Terence Partridge, Miranda Grounds and John Sloper showed that donor cells could fuse with host cells or myofibres in adult muscle, following grafting of minced muscle between mice with different isoenzyme subtypes [89,90].

Although likely that most donor-derived myonuclei in host tissue were formed from labelled myoblasts, other cells in the grafted donor muscle tissue, including endothelial cells and endomysial fibroblasts, could also have contributed. To circumvent this possibility, Bruce Lipton and Schultz enzymatically dissociated cells from muscle of juvenile or adult quail, or juvenile rat, and expanded the (satellite cell-derived) myoblasts *in vitro *while labelling with tritiated thymidine. Pelleted myoblasts reimplanted in muscle of the original donor produced myonuclei within host myofibres, but there was no labelled fibrogenic, endothelial or other non-muscle cell types. Implantation of myoblasts under the skin also resulted in myotube formation [91], implying that satellite cell-derived myoblasts retained their myogenic state *in vivo*, irrespective of whether they were transplanted into a muscle, or non-muscle, environment.

By the time that the satellite cell had come of age at 18 therefore, its basic role in muscle function had been elucidated; that is, satellite cells were the myogenic precursor cells able to supply myoblasts for postnatal muscle growth and regeneration (Figure 1). Contemporary reviews of the early 1980s give the then perspective [92,93], as does the 1979 book of conference proceedings, *Muscle Regeneration*, edited by Mauro [94].

### Fast forward to the present

These fundamental studies provided the groundwork for further advances, some of which are briefly highlighted here. The methods of Bischoff and Konigsberg largely produced myofibre fragments [83,84], and isolation of intact muscle fibres had required fixation [95,96]. Muscle fibres/fragments could be obtained using collagenase [97], and Anne Bekoff and William Betz combined collagenase digestion and trituration to obtain complete viable muscle fibres [98], which could be maintained in culture [99]. Interested in acetyl choline sensitivity, Bekoff and Betz did not specifically look for satellite cells although they were still present [100]. Analysis of satellite cells associated with isolated myofibres was optimised and championed by Bischoff in a series of papers through the 1980 and 1990s, investigating their activation and proliferation (e.g. [101]). Examination and manipulation of satellite cells retained in their niche on an isolated myofibre (Figure 4) has now become a cornerstone of many studies, and detailed protocols for myofibre isolation are available [102,103].

The need for electron microscopy to confirm satellite cell identity had long been a limitation, and was gradually replaced by molecular markers, allowing their identification at the light level. Discovery of the myogenic regulatory factor family (Myf5, MyoD, myogenin and MRF4) in the late 1980s was a seminal moment in understanding the specification of the myogenic lineage and control of muscle development [104]. Crucially, these same genes were shown to be redeployed by myoblasts in regenerating muscle [105-107], so linking the genetic control of developmental and regenerative myogenesis. These genes also provided important new molecular markers for satellite cell-derived myoblasts [105-107]. It was not until 1994 though, that the first useful marker of quiescent murine satellite cells was described (M-cadherin) [108], with an increasing number since reported [109,110], of which Pax7 [111] probably remains the most practical and convenient (Figure 4). Antibodies against cell surface antigens of satellite cells including CD34 [112] and alpha-7 integrin [113] have proved valuable as part of a cohort of markers for isolation of muscle stem cells using fluorescent activated cell sorting (FACS). In addition to permitting myogenic progression to be monitored, molecular markers have also allowed the *in vitro *'reserve' cell model of myogenic cell self-renewal to be developed in the 1990s [114-116]. The subsequent demonstration of the role of Pax7 in satellite cell function [108] facilitated more sophisticated *in vitro *models of self-renewal using plated cells [117,118] or in combination with culture of satellite cells retained on isolated myofibres [119].

Detailed autoradiographic studies *in vivo *defined the kinetics of myoblast proliferation during mammalian regeneration after various types of injury [120,121]. Lineage tracing with tritiated thymidine however, had a number of drawbacks including label dilution [122], and was replaced in large part, by mouse models. Mice with different isoenzyme subunits had been used to show that donor cells fused to form hybrid myofibres after grafting into adult [89,90], which also indicated that satellite cell/myoblast transplantation may be a therapy for muscle disease [123] and led to a series of human trials (reviewed in [110]). Mutant and genetically modified mice also provided a powerful tool for both identifying satellite cells [112,124,125], and for lineage tracing to the single cell level, for examination of myogenic potential and contribution to the satellite cell pool [125-128]. Such genetic tools enabled the satellite cell to be classified as a stem cell. Grafting a single myofibre together with its associated satellite cells resulted in the generation of many new satellite cells in the host muscle, so demonstrating their ability to self-renew: requisite for a stem cell [128]. Indeed, self-renewal was later shown after transplanting just a single murine satellite cell [129].

This increased sensitivity of lineage tracing also allowed the age-old hypotheses on the source of cells for muscle regeneration to be revisited. Zenker [2] and Waldeyer [3] both thought that myogenic precursors arose from the connective tissue of muscle. To date, various cells with myogenic potential have been described that reside in the connective tissue including Sk-34 [130], PW1^+^/Pax7^- ^interstitial cells (PICs) [131], and maybe also side population [132], with others, such as mesangioblasts, found associated with the vasculature [133]. Maslowsky [17] first proposed that cells from the circulation could form muscle, with tritiated thymidine studies of the 1960s coming to the same conclusion [134]. Circulating cells with myogenic potential have now been characterised, such as AC133(+) stem cells [135]. Bone marrow was proposed as the source of these circulating cells, but grafting bone marrow into muscle did not result in readily measurable amounts of new muscle being generated [136]. However, use of a sensitive *nlacZ *transgene did allow the detection of rare donor-derived myonuclei [126]. The inherent myogenic potential of cells responsible for such 'unorthodox' myogenesis is questionable, with most expressing muscle genes only after undergoing myogenic reprogramming following interaction/fusion with myoblasts or myofibres (for example, [137]). This begs the question of whether they have a physiological role in muscle regeneration, or are merely noise in the system, revealed by the sensitivity of the techniques employed.

Finally, myonuclear dedifferentiation was long thought to occur in amphibians [60,138,139], but this has recently been questioned by the description of satellite cells in salamander [140,141]. Also proposed to occur in mammals until the early 1970s [142], dedifferentiation of myonuclei can still not be totally discounted as a possible source of myoblasts for muscle regeneration [143,144].

## Conclusions

### Known knowns, known unknowns and unknown unknowns

So what do we know after 50 years of research into muscle satellite cells? Essentially, that they are resident muscle stem cells, responsible for supplying myoblasts for skeletal muscle growth, homeostasis, hypertrophy and repair. Of the many known unknowns, little is established about how satellite cells are maintained in a quiescent state [145,146], while how they are then activated to enter the cell cycle is beginning to be unravelled, with signalling pathways including Notch/Delta clearly implicated [147]. However, a relatively new mode of satellite cell control is gene regulation via miRNA, and evidence is beginning to accumulate of their role [148,149]. Similarly, the sublaminal satellite cell niche is not well characterised, but recent work on modelling the niche in culture is already providing useful insight [150,151].

There is much debate about whether the satellite cell population is heterogeneous [152]. Satellite cell properties vary depending on their muscle of origin, but even within the same muscle, can be subdivided using genetically modified mice [125,153] and are functionally heterogeneous *ex vivo *[124,154,155]. Different regenerative potentials have been ascribed to satellite cell subpopulations isolated by FACS using various antigen combinations [153,156]. However, it is often difficult to confirm the provenance of these subpopulations *in vivo*, as some of the antibodies used for FACS are not effective for immunocytochemistry. If these functional differences are related to heterogeneity within the sublaminal niche, is the satellite cell population composed of lineage-based satellite 'stem' cells and myogenic precursors [153]? Or do satellite cells evolve into a continuum of cells with more, or fewer, stem cell characteristics, perhaps because some cells have undergone fewer divisions [157]? However, resolution of this awaits prospective markers able to identify satellite 'stem' cells, as has happened recently for the gastrointestinal tract [158]. Whatever the nature of the satellite cell pool, we still need to better understand what dictates which progeny ultimately undergoes self-renewal and which differentiates? Recent publications implicate Wnt signalling, with cell fate being related to the plane of cell division with respect to the myofibre [159].

By definition, unknown unknowns are rather hard to predict, but often prove to be the most exciting. A recent example is the revision of the role of Pax7 in adult muscle, long thought essential for satellite cell function [111]. Inducible Cre-mediated inactivation of the *Pax7 *locus was used to demonstrate that Pax7 was necessary for satellite cell function during postnatal muscle growth in mouse. Surprisingly however, when the *Pax7 *locus was inactivated in satellite cells in adult, muscle regeneration was unaffected [160]. These observations have prompted reappraisal of a hitherto central tenet of the transcriptional control of the satellite cell.

Arguably the pre-eminent unknown unknown in regenerative myogenesis of the modern era was the existence of the satellite cell, whose discovery by Mauro and Katz in 1961, gradually created a paradigm shift in our understanding of muscle biology. Widely regarded as the major source of myonuclei for skeletal muscle growth and repair, the description of other muscle precursor cells had cast some doubt on the importance of the satellite cell. However, several upcoming studies detailing the lack of significant muscle regeneration after genetic ablation of the satellite cell pool further confirm their indispensable role in repairing skeletal muscle (e.g. [161]). Hopefully the next 50 years of research into satellite cells will prove as enthralling as the first 50 have!

## Competing interests

The authors declare that they have no competing interests.

## Authors' contributions

Both authors read and approved the final manuscript. While both authors contributed to the writing of this manuscript, JS read and mainly reviewed the manuscripts published in German.
